# Lifting capacity prediction model using physical performance measures among construction workers

**DOI:** 10.1038/s41598-022-05106-0

**Published:** 2022-01-20

**Authors:** Sidhiprada Mohapatra, Aparajita Verma, N. Girish

**Affiliations:** Department of Physiotherapy, Manipal College of Health Professions, Manipal Academy of Higher Education, Manipal, Karnataka India

**Keywords:** Diseases, Risk factors

## Abstract

Manual materials handling is performed in many workplaces and is a significant risk factor for musculoskeletal injuries. The identification of lifting capacity is important to reduce the occurrence of musculoskeletal injuries. Lifting capacity is difficult to evaluate at the workplace. Therefore, there is a need to develop an alternate method that is easy and could be performed at the workplace. The study aimed to develop a lifting capacity prediction model for construction workers based on muscle strength and endurance. In this study, 65 construction workers were recruited; their socio-demographic and physical characteristics like core strength and endurance, grip strength, and lower limb flexibility were assessed. The lifting capacity was assessed using progressive isoinertial lifting evaluation. Stepwise multiple linear regression was carried out to develop the prediction model. The study suggested that age, BMI, grip strength, flexibility, prone plank, and trunk lateral flexor endurance tests have significantly influenced lifting capacity. Hence prediction model is developed using these variables. The regression model developed would help in easy estimation of lifting capacity among construction workers, which could be even administered with minimal skills by site supervisors or managers. It might help in the decision-making during pre-placement or return to work evaluations, thereby minimizing the incidence of low back disorders.

## Introduction

Occupation-related low back disorder is the most common and most costly musculoskeletal disorder experienced in the workplace^1^. World Health Organisation has recognized Lower back disorders (LBD) as one of the top three occupational health problems worldwide^2^. The prevalence rate of LBD is higher among construction workers which is 68% followed by workers in the manufacturing industries which is 49.9%^3^. In India, more than 75% of the construction workers report LBDs^4^, and Manual Materials Handling (MMH) activities are considered to be the important contributor to the etiology of LBDs^5^. The amount of load a person can lift has been identified as the major independent risk factor for LBDs; hence determining lifting capacity is important in minimizing its incidence^6^. Lifting capacity evaluation is performed using isometric, isokinetic, and isoinertial methods, among these isoinertial, is considered to be the best one as it is safe, inexpensive, simple, and dynamically replicates the real-life lifting^7,8^.

Most often lifting capacity evaluation is not performed because of the complexity involved in the existing lifting capacity evaluation in terms of time, lab, expensive equipment, and expertise required to perform it^9^. So the workers in the construction field are most often forced to lift weights beyond their physical capabilities^10^. Hence there is a need to develop a simple model for predicting lifting capacity, which can be done with minimal skills by site supervisors or managers. There are a few models available, either to estimate a Recommended Weight Limit (RWL) or to predict maximum lifting weight^11–13^. However, these models have limitations as the dynamic joint strength data were not included in the models, these models are not free for industries and most of these models are not considering the physical performance characteristics of the person who is involved in MMH. The objective of this study was to develop a regression model for predicting lifting capacity among construction workers.

## Methods

### The experimental approach to the problem

The study was initiated after obtaining clearance from the Institutional Ethics Committee, Kasturba Hospital, Manipal (IEC: 163/2019), it was registered under the Clinical Trials Registry, India (CTRI/2019/05/026189) and it was conducted between November 2019 and April 2020. A verbal advertisement about the study at the construction sites was done and the study was carried out at randomly selected construction sites in Manipal and Exercise therapy lab, Department of Physiotherapy, Manipal College of Health Professions, Manipal. This study adopted an experimental design, male construction workers with a minimum of one year of work experience and without any acute illnesses in the past 6 weeks participated in this study. A wooden box, made of compressed wood of dimensions 48 × 36 × 24 cm and 4.5 kg weight with a semilunar shaped cut-out handle 5 cm below the top border on the longer side was prepared after reviewing the literature^14^. The procedure was explained and demonstrated to the workers. The dependent variable evaluated was the lifting capacity in kg and their socio-demographic and physical characteristics like core strength and endurance, grip strength, and lower limb flexibility were the independent variables. The sequence in which the tests were carried out was randomized using a lottery method to avoid order-effect bias.

### Subjects

The workers who showed interest to participate were requested to sign a written informed consent or a thumb impression was taken from uneducated workers in the presence of a witness. A sample size of n = 65 was estimated based on an effect size of 0.15, an alpha error of 0.05, a power of 0.95, and an inclusion of 3 or more predictors, as reported by Gregory and Daniel 2007 in “Sample Size Recommendations at Selected Levels of Squared Population Multiple Correlation Coefficients for Varying Numbers of Predictor Variables”^15^.

### Procedure

Baseline parameters like age, body weight, height, and resting heart rate (using polar HR monitor, FTI, Finland) after making them lie in the supine position for five minutes were recorded. Instructions related to the performance of Progressive Isoinertial Lifting Evaluation (PILE) and termination criteria were given to all the participants before commencing the test^8^. The participants were instructed to lift the box when the investigator said ‘start’ and a stop-watch also was started simultaneously. Each participant was made to perform PILE from floor to shoulder level using a self-selected lifting technique.

The participants were advised to lift the box from the floor to the shoulder and lower it back to the floor 4 times in 20 s intervals. A load increment of 4.5 kg was added every time they completed the stage. The participants continued lifting performance until they terminated the protocol because of any of the reasons including fatigue, discomfort, and shortness of breath, inability to complete 4 lifts, lift a load of 60% of their body weight or achieve 85% of their age determined heart rate. Immediately after terminating the protocol, they were asked to lie down in a supine position on a couch for a minimum of five minutes, and the termination criteria were recorded during the first minute of HR recovery. The parameters which were recorded were the stage and increments of PILE and weight lifted in kg^8^.

The physical performance measures evaluated were flexibility using sit and reach test, strength using handgrip strength test, and endurance using a prone plank, trunk flexor, extensor, and lateral flexor endurance tests. These tests were selected as they are found to have a relation with the lifting capacity based on literature review and expert consensus, also they are easy to perform in the real workplace. For the sit and reach test, the participants were made to sit with the knees extended, feet resting against a box, and were instructed to position one hand on top of the other with palms facing down. They were asked to lean as forward as possible along the measurement scale without flexing the knees and the furthest distance reached along the scale was recorded to the nearest 0.5 cm^16^. The handgrip strength was assessed using the JAMAR hand dynamometer for which the participants were made to sit in an armless chair, with the elbow flexed to 90 degrees. The dynamometer was held in the dominant hand and participants were asked to press and grip it to their maximum strength for about 5 s. Three readings were taken and the average was calculated^17^.

The prone plank test evaluates the trunk stability wherein the participants were positioned prone with forearms placed below the chest and the elbow positioned at a 90-degree angle. In this timed test, the participant had to raise the pelvis from the floor and maintain a flat position and the test was terminated when the participant was not able to hold the position^18^. In the trunk flexor endurance test the participants were timed in a semi-reclining position with the hips and knees at 90 degrees. They were instructed to keep the arms across the chest with both hands touching the shoulder of the opposite side. The participants had to lean beside a board that is kept in an incline position at a 60-degree angle, the participant has to maintain the head in a neutral position. They had to hold this position after the board was removed (moved 4 inches back) by involving the abdominal muscles to sustain a flat-to-neutral spine, without arching the back during the test. Any evident changes in the position of the trunk like rising in the low-back arch or an aberration from the neutral position terminated the test protocol^19^.

In the trunk extensor endurance test, the participants were made to lie prone, the lower legs were stabilized or fastened to the table using a strap with the iliac crests positioned at the edge. Initially, participants supported the weight of the upper body on the arms of a chair and they were instructed to lift the trunk at the level of the legs with arms flexed in a crossed position on the chest. The goal of the test was to sustain a horizontal prone position for the longest duration possible. When the participant fell below the horizontal position or on the development of trick movements the test was stopped^20^.

The trunk lateral endurance test or the side-bridge test evaluated the endurance of the lateral core muscles. The participants were on the side of his body, with both the legs extended and the feet one in front of the other. The elbow of the supporting arm (the arm which is on the lower side while the side-lying) was placed below the shoulder with the forearm facing out and the other upper limb resting along the side of the body. They were instructed to take up to the full side bridge position by raising the hip, the trunk supported only by foot, and the elbow/forearm of the lower arm of the participant. The goal of the test was to sustain this position for the longest duration possible and any evident changes in the position of the trunk, aberration from the neutral spine or the hips shift backward or forward terminated the test protocol^21^.

### Statistical analyses

SPSS version 16.0 for windows (SPSS Inc. in Chicago) was used to analyze the data. Descriptive statistics were reported using means, standard deviations, frequencies, and/or percentage distributions as appropriate for the type of data collected. Log transformation was done as the data was not following linearity. Univariate and multivariate stepwise linear regression was used to examine the relationship between the candidate predictors and the outcome variable and to develop the prediction model. The stepping criterion for entry into the model was based on the significance level of the *F*-value ≤ 0.050. The level of significance was set at *p* ≤ 0.05.

### Ethics approval

Ethics approval was taken from the institutional ethics committee, Kasturba hospital, Manipal (IEC: 163/2019), Kasturba Hospital, and CTRI registration (CTRI/2019/05/026189).

### Consent to participate

All procedures followed were in accordance with the ethical standards of the responsible committee on human experimentation (institutional and national) and with the Helsinki Declaration of 1975, as revised in 2000. Informed consent was obtained from all patients for being included in the study.

## Results

**Figure 1:**
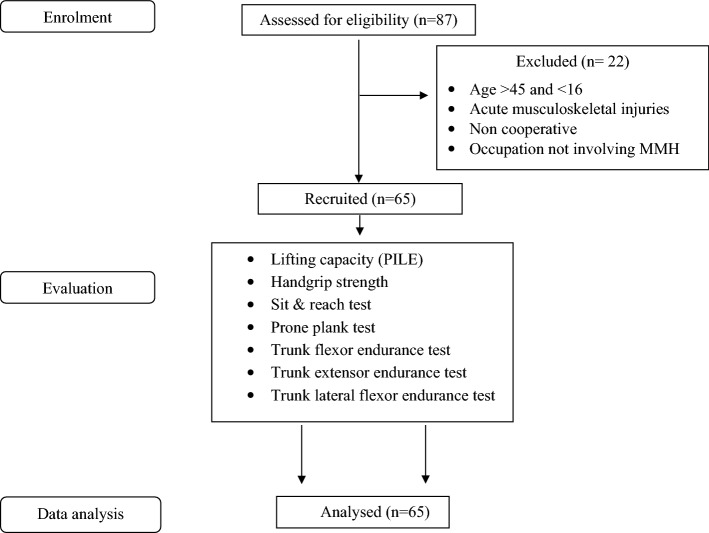
Flow of participants.

The flow of participants is represented in Fig. 1, out of 87 workers screened, 65 workers were included in this study and 22 workers were excluded due to various reasons mentioned. The socio-demographic characteristics, lifting capacity, and physical performance measures of the construction workers are depicted in Table 1 (see Table 1). The mean age of the participants was 27.9 ± 8.17 and only 10.76% (n = 7 out of 65) of participants were above 40 years. The BMI of the participants ranged from 15.73 to 26.47 kg/m^2^ with 23 participants in the overweight category (> 25 kg/m^2^), 5 in the underweight category (< 18 kg/m^2^), and 37 in the healthy range of BMI (18–25 kg/m^2^). The mean resting heart rate of the participants was 77.64 ± 8.05 beats/min and it ranged from 50 to 92 beats/ min. The flexibility measured by the sit and reach test ranged from -15.00 to 14.50, with 16 workers in the negative range indicating poor flexibility and 6 workers overshooting the fingers over the toes showing hyper-flexibility. Some participants were unable to perform prone plank (n = 1), trunk flexor (n = 3), trunk extensor (n = 3), and trunk lateral flexor (n = 2) and have been scored zero in these endurance tests.

**Table 1:** Socio-demographic characteristics, lifting capacity, and physical performance measures (n = 65).

Variables	Mean ± SD
Age	27.9 ± 8.17
BMI (kg/m^2^)	20.98 ± 2.31
Hours of work per day	10.6 ± 2.25
Resting heart rate (beats/min)	77.64 ± 8.05
Handgrip strength (kg)	37.6 ± 7.36
Sit and reach (cm)	-1.5 ± 4.81
Prone plank (sec)	22.04 ± 18.93
Trunk flexor endurance test (sec)	34.12 ± 41.86
Trunk extensor endurance test (sec)	21.57 ± 31.61
Trunk lateral flexor endurance (sec)	14.89 ± 12.41
Lifting capacity (kg)	22.8 ± 5.57

Variables	Number (%)
Trained	3 (4.6)
**Category of work**	
Carpenter	12 (18.5)
Mason	5 (7.7)
Electrician	4 (6.2)
Multipurpose	44 (67.7)
**Substance abuse**	41 (63.1)
Smoking	4 (6.2)
Alcohol	4 (6.2)
Pan	2 (3.2)
Gudka	2 (3.2)
Multiple	29 (43.1)
Comorbidities	1(1.5)
**Literacy level**	
Illiterate	7 (10.8)
Primary school (< 8th standard)	17 (26.2)
High school (8–10th standard)	30 (46.2)
PUC (11th and 12th standard)	8 (12.3)
Degree (> 12th standard)	3 (4.6)
**Work experience**	
6 month–1 year	5 (7.7)
1–5 years	29 (44.6)
More than 5 year	31 (47.7)
**Musculoskeletal discomfort**	
Nil	43 (66.2)
Neck discomfort	3 (4.6)
Shoulder discomfort	1(1.5)
Wrist/hand discomfort	1(1.5)
Upper back discomfort	1(1.5)
Lower back discomfort	8 (12.3)
Hip/thigh discomfort	1(1.5)
Knee discomfort	2 (3.1)
Ankle discomfort	5 (7.5)

Table 2 depicts the univariate and multivariate analysis of demographic characteristics and physical measures of the workers with the lifting capacity (see Table 2).

**Table 2:** Factors analyzed while developing lifting capacity prediction model (n = 65).

Variables	Univariate analysis			Multivariate analysis		
	B	*p*-value	*r* value (95%CI)	B	*p*-value	*r* value (95% CI)
Age	− 0.125	0.145	0.183 (− 0.293, 0.044)	− 0.221	**0.034**	− 0.183 (− 0.424, − 0.018)
BMI	0.223	0.463	0.093 (− 0.381, 0.826)	0.828	**0.022**	0.193 (0.122, 1.533)
RHR	0.074	0.395	0.107 (− 0.099, 0.248)	− 0.001	0.997	0.107 (− 0.171, 0.168)
Substance abuse	1.247	0.388	0.109 (− 1.621, 4.115)	1.495	0.308	0.109 (− 1.420, 4.410)
Musculoskeletal discomfort	− 0.097	0.641	0.059 (− 0.511, 0.317)	0.196	0.320	− 0.059 (− 0.273, 0.665)
Grip strength	0.264	**0.004**	0.353 (0.088, 0.441)	0.148	**0.002**	0.353 (0.051, 0.348)
Sit and reach	0.155	0.309	0.128 (− 0.147, 0.457)	0.230	**0.048**	0.173 (0.127, 0.586)
Prone Plank	0.081	**0.026**	0.276 (0.010, 0.152)	0.025	**0.005**	0.316 (0.124, 0.174)
Trunk flexor endurance	0.042	**0.010**	0.316 (0.010, 0.074)	0.020	**0.013**	0.276 (0.025, 0.066)
Trunk extensor endurance	0.034	0.129	0.190 (− 0.010, 0.077)	− 0.016	**0.046**	0.190 (0.061, 0.092)
Trunk lateral flexor endurance	0.153	**0.008**	0.327 (0.042, 0.264)	0.102	**0.005**	0.328 (− 0.070, 0.274)

Significant values are in bold.

### Model development

As seen in Table 2, grip strength, prone plank, trunk flexor endurance, and trunk lateral flexor endurance time have shown a significant relationship with the lifting capacity in univariate analysis. But when stepwise multivariate analysis was carried out, age, BMI, grip strength, flexibility (sit and reach), core stability (prone plank), and trunk lateral flexor endurance test showed a statistically significant relationship with the lifting capacity. Among these variables, only age showed a significant negative correlation whereas all the other variables had a significant positive relationship with the lifting capacity, hence these variables were taken to develop the regression equation. A significant regression equation was found (F (11, 54) = 2.094, *p* < 0.040) with an *R*^2^ of 0.646.

$$\begin{gathered} {\text{Lifting}}\,{\text{capacity}}\,{\text{in}}\,{\text{kg}} = 3.177 - \left[ {0.228({\text{age}}) + 0.868({\text{BMI}}) + 0.193({\text{grip}}\,{\text{strength}})} \right. \hfill \\ \left. { + 0.270({\text{flexibility}} + 0.204({\text{prone}}\,{\text{plank}}\,{\text{time}}) + 0.165({\text{trunk}}\,{\text{lateral}}\,{\text{flexor}}\,{\text{endurance}}\,{\text{time}})} \right] \hfill \\ \end{gathered}$$

Where age is measured in years, BMI in Kg/m^2^, grip strength in kg, flexibility measured by sit and reach test in cm, core stability measured by prone plank test in seconds, and trunk lateral flexor endurance test measured in seconds.

## Discussion

This study aimed to develop a model for the prediction of lifting capacity among Indian construction workers. Lifting capacity is assessed during pre-placement and return to work process for prescribing recommended weight among workers who are involved in MMH activities. Since the protocols followed for lifting capacity evaluation are tedious, time and space consuming, and requirement of trained personnel, it is rarely performed at the workplace. Hence, the model developed in this study may be used as an alternate method thereby addressing the limitations of the existing protocols. These existing models have some limitations as the dynamic joint strength data were not included in the models, these models are not free for industries and most of these models are not considering the physical performance characteristics of the person who is involved in MMH^11–13^. Eleven socio-demographic characteristics and physical performance measures were considered as independent variables. The age of the workers in this study ranged from 18 to 45 years. This age range was purposively kept in the selection criteria, as 18 years was the legally permissible age to work in India and the upper limit was decided to avoid the influence of age-related musculoskeletal problems on lifting capacity. As hypothesized, age was shown to have a negative relation with the lifting capacity. Girish et al. 2018 have studied the influence of age on lifting capacity and they have reported that the lifting capacity will be maximum during the 3rd and 4th decade and a gradual decline afterward^22^.

BMI and lifting capacity was shown to have a positive correlation in the current study. A relationship of body weight and lifting capacity of r = 0.63 has been reported by Gross et al. 2000^23^. Bodyweight and BMI are directly related, this explains the positive relationship between BMI and lifting capacity found in this study.

A positive relationship between grip strength and lifting capacity has been established in this study. Grip strength is recognized as a global indicator of overall muscle strength and as a predictor of various adverse health events^24^. Lifting is an activity involving many muscle groups and the entire kinetic chain of the human body. Since grip strength indicates overall muscle strength, it was hypothesized that there will be a strong correlation between grip strength and lifting capacity, however a weak to a moderate relationship only was found. This relationship would have been better if the evaluation was adopted using a static lifting dynamometer which is isometric in nature.

Flexibility measured by sit and reach test and lifting capacity was found to have a positive relationship. In this study, 16 workers had negative readings in the sit and reach test which indicates a considerable amount of muscular tightness. A stratified analysis showed that for those who had muscle tightness (n = 16), the mean lifting capacity was 21.06 ± 5.31, whereas it was 22.50 ± 4.02 for those who did not have a tightness (n = 6). A semi-squat lifting technique was adopted by a majority of the participants. It was presumed that some amount of muscular tightness helps produce adequate muscle contractility and torque, thereby generating good power. But this may not hold with lifting capacity as indicated in this study^25^.

The core muscle strength and endurance evaluated by the prone plank test and trunk lateral flexor endurance test observed a positive relationship with lifting capacity and it appeared as a significant contributor in the regression model. In healthy individuals, the deep or core muscles, such as multifidus (which attaches directly to the lumbar vertebrae) and transversus abdominis (which attaches indirectly to the lumbar vertebrae via the thoracodorsal fascia) is responsible for providing segmental spinal stabilization^26^. The prone plank position is found to activate those muscles; the stronger the core muscles, the better would be the lifting capacity^27^.

Pinder and Boocock 2014 have published a paper on the prediction of the maximum acceptable weight of lift from the frequency of lift, however that study has not considered the physical characteristics of the workers^28^. To the best of our knowledge, this is the first study that has considered physical performance measures in developing a regression model for determining lifting capacity.

This study has a few limitations. Firstly, lack of blinding during the evaluation, secondly the physical activity status of the workers were not monitored, and thirdly, the study was carried out after the working hours, hence fatigue among the workers would have influenced the results. Also, validation of the developed model was not conducted in the present study, which is the fourth limitation. As a future scope, studies are needed to model the requirements of the frequency of lift, duration of lifting efforts, variety of hand-object coupling, and combined lifting and reaching. Further studies are needed to validate the present model to test its accuracy and generalisability. Also, objective evaluation of physical activity status using wearable devices or activity monitors need to be used in future studies.

## Conclusion

Age, BMI, grip strength, flexibility, prone plank, and trunk lateral flexor endurance tests have a significant influence on lifting capacity. Hence prediction model is developed using these variables.

### Recommendations

The regression model developed would help in easy estimation of lifting capacity among construction workers, which could be even administered with minimal skills by site supervisors or managers. It might help in the decision-making during pre-placement or return to work evaluations, thereby minimizing the incidence of low back disorders.

## Data Availability

The datasets analyzed during the current study are available from the corresponding author on reasonable request.
